# Initial Perspectives From Rural-Residing Adults on a Digital Cognitive Health Coaching Intervention: Exploratory Qualitative Analysis

**DOI:** 10.2196/51400

**Published:** 2024-07-22

**Authors:** Jennifer Rae Myers, Kelsey N Bryk, Erica N Madero, Jacob McFarlane, Anthony Campitelli, Joshua Gills, Megan Jones, Sally Paulson, Michelle Gray, Jordan M Glenn

**Affiliations:** 1 Neurotrack Technologies Redwood City, CA United States; 2 Exercise Science Research Center Department of Health, Human Performance and Recreation University of Arkansas Fayetteville, AR United States; 3 St. Elizabeth Healthcare Clinical Research Institute Edgewood, KY United States

**Keywords:** Alzheimer disease, cognition, intervention, rural issues, digital health, geriatric, geriatrics, elder, elderly, diabetes, diabetes mellitus, dementia, digital cognitive health coaching, rural, countryside, qualitative study, thematic analysis, mHealth, telehealth, health informatics, mental health, behavioral change, healthy lifestyle, coach support, self-awareness, prevention

## Abstract

**Background:**

A growing body of research has examined lifestyle-based interventions for dementia prevention. Specifically, health coaching interventions have been linked to decreased risk of Alzheimer disease (AD) comorbidities, such as diabetes. Despite the association, there is a lack of research examining the efficacy and perception of digital health coaching on reducing AD risk. Understanding the perceived benefits of participating in a digital health coach program is critical to ensure long-term use, including participant adherence and engagement.

**Objective:**

The purpose of this study is to examine the initial attitudes toward a digital health coaching intervention aimed at preventing cognitive decline among at-risk, rural participants.

**Methods:**

This exploratory qualitative study is part of the ongoing Digital Cognitive Multidomain Alzheimer Risk Velocity Study (DC-MARVel; ClinicalTrials.gov NCT04559789), a 2-year randomized control trial examining the effects of a digital health coaching intervention on dementia risk, cognitive decline, and general health outcomes. Participants were recruited from the northwest region of Arkansas via word of mouth, email, local radio, and social media. At the time of the analysis, 103 participants randomly assigned to the health coaching group completed an average of 4 coaching sessions over a 4-month period. The intervention included asynchronous messages 1-2 times per week from their health coach that contained health education articles based on the participant’s goals (eg, increase physical activity), unlimited access to their coach for questions and recommendations, and monthly meetings with their coach via videoconference or phone to discuss their goals. Participants were asked 2 open-ended questions, “What were your top 1 or 2 takeaways from your recent Health Coaching session?” and “Is there anything you would change about our Health Coaching sessions?” A thematic analysis was conducted using feedback responses from 80 participants (mean age, SD 7.6 years).

**Results:**

The following four themes emerged from participants’ feedback: (1) healthy lifestyle and behavioral changes, (2) a sense of self-awareness through introspection, (3) value in coach support, and (4) a desire for a change in program format (eg, frequency). In total, 93% (n=74) of participants expressed that the intervention needed no changes.

**Conclusions:**

Initial participation in the digital cognitive health coaching intervention was well received, as evidenced by participants reporting value in goal setting and strategies for healthy lifestyle and behavioral changes as well as self-reflection on their personal lifestyle choices. Feedback about their assigned coach also offers insight into the importance of the coach-participant relationship and may serve as a significant factor in overall participant success. Given the exploratory nature of this study, more robust research is needed to elicit more information from participants about their experiences to fully understand the acceptability of the digital health coaching intervention.

**Trial Registration:**

ClinicalTrials.gov NCT04559789; https://clinicaltrials.gov/show/NCT04559789

**International Registered Report Identifier (IRRID):**

RR2-10.2196/31841

## Introduction

### Background

By the year 2030, it is estimated that 78 million people will be living with dementia, a drastic increase from the 55 million people today [1]. Individuals living with Alzheimer disease (AD) experience various cognitive, behavioral, psychological, and physical changes leading to difficulty completing activities of daily living, which in turn may require a patient to take a constellation of expensive medications and therapies. The estimated cost of dementia to the US economy in 2019 was US $244 billion [2]. By 2050, it is estimated that the total annual health care cost to an individual with AD in the United States will be US $140,012, adding up to a total annual cost of US $1.5 trillion [3]. Some interventions (eg, medical, pharmaceutical, and lifestyle) may delay the onset of AD by 5 years, reducing AD prevalence in 2050 by 41%, with a subsequent 40% reduction in costs, saving an individual over US $500,000 in medical bills over their lifetime [3]. However, it remains to be determined which intervention, or combination thereof, is most beneficial to an individual at risk of developing AD.

Over the years, several risk factors of AD have been identified, with much research focusing on genetics. In conjunction, the production of medical and pharmaceutical interventions is at the forefront of AD research; however, both the scientific and clinical community continue to face challenges when achieving success with these interventions, as the success rate of AD drugs remains low at 0.4% [4]. Although genetic factors are associated with a higher risk of developing dementia, such as the apolipoprotein E (APOE) genotype, recent research has shown that an individual’s lifestyle plays a significant role in the risk of developing AD [5]. Several lifestyle-related and modifiable risk factors for dementia have been identified, which account for an estimated 40% of worldwide dementia cases. These risk factors include physical inactivity, depression, infrequent social contact, excessive alcohol consumption, and smoking [6,7]. The identification of these risk factors has led some researchers to investigate the possibility of decreasing AD risk by modifying these lifestyle habits in adults deemed to be at risk of dementia. A recent study demonstrated individuals with a higher genetic risk, but favorable lifestyle habits are less likely to develop dementia than individuals with the same high genetic risk but with a less favorable lifestyle [8]. These findings suggest that even individuals with a higher genetic risk are able to reduce their risk of developing dementia long before any symptoms arise. Research such as this has laid the groundwork for lifestyle-based dementia prevention interventions, and although this field of research is still at an early stage, a few studies have demonstrated promising results in preventing dementia [9-14].

### Multidomain Lifestyle Interventions for Cognitive Decline

Currently, there is no gold standard intervention for dementia prevention; however, lifestyle interventions have been shown to reduce cognitive decline in older adults. The FINGER study (Finnish Geriatric Intervention Study to Prevent Cognitive Impairment and Disability) was a 2-year randomized controlled trial that demonstrated the efficacy of a multidomain lifestyle intervention for older adults at risk of cognitive decline [9]. In this study, the intervention group received individualized coaching sessions on nutrition, exercise, cognitive training, and regular monitoring of their vascular health, and after 2 years, this group demonstrated improved cognitive function, supporting the notion that a multidomain lifestyle intervention could help to improve cognitive functioning in adults who are at risk of cognitive decline [10]. Although the FINGER study has yielded convincing results for lifestyle changes and dementia prevention, a limitation of this study was the clinic-dependent approach, requiring all participants to commute to and from a local clinic for all intervention sessions and study assessment appointments. This approach to intervention has limitations for individuals who do not have access to health care resources or reliable transportation. These limitations are already barriers for individuals who are of a lower socioeconomic status, a known risk factor for dementia, as well as individuals who live in rural communities [15-17]. Internet-based interventions are feasible and allow for a more accessible and more cost-effective way for a greater population of at-risk individuals to participate in lifestyle intervention programs. Participants may struggle with adherence to a lifestyle intervention when they are to be self-guided through the intervention; therefore, one additional feature of these lifestyle intervention programs is lifestyle coaching for guidance [11]. Using a health coach for guidance through an individualized and digital health program may help at-risk individuals to adapt to new lifestyle habits and adhere to the program, in turn, creating long-term lifestyle changes.

Several clinical studies in urban centers have used individualized lifestyle interventions that involve coaching sessions, with positive feedback from the participants [11-14]. Older adults who completed a 52-week study involving virtual coaching sessions on physical activity, nutrition, and other lifestyle habits reported a high level of satisfaction with the virtual coaching program as well as improvements in their exercise, eating, and sleep habits [14]. While research shows these lifestyle interventions are beneficial to individuals living in urban areas with an abundance of resources, such as health care and reliable transportation, the question becomes about the feasibility of these intervention programs for rural communities. The lack of access to internet services has previously been identified as a barrier within rural communities, although with recent technological advances, recent research shows approximately 72% of rural residents in the United States have access to broadband internet services and 80% of rural residents have a smartphone, similar to the 77% and 89% of urban participants, respectively [18]. More specifically, healthy rural residents identified that personal technology is feasible and desirable for a digital health coaching (HC) program. However, for a program to be successful, it should aim to use the community’s, not outside, resources [19]. These virtual interventions involving one-on-one coaching sessions are particularly useful to individuals living in rural communities, where a lack of access to health care, reliable transportation, and other lifestyle resources are common barriers for these communities [20]. With recent technological advances and greater availability, virtual health care may also benefit rural residents who are at risk of developing dementia, specifically digital dementia prevention intervention involving individualized lifestyle coaching.

Understanding the thoughts and feedback of rural participants on a digital lifestyle program involving one-on-one coaching sessions will help to further understand the potential feasibility of this style of program administration, as well as ways to modify or improve the administration methods (eg, frequency) and the coaching itself. As adherence and engagement are crucial to the success of digital HC, it is important to explore how individuals who participate in digital HC programs perceive its benefits. This study explores the initial attitudes and acceptability of a digital cognitive HC program among rural-residing individuals at risk for AD.

## Methods

### Intervention Setting

This exploratory project is part of the ongoing Digital Cognitive Multidomain Alzheimer Risk Velocity study (DC-MARVel; NCT04559789) [12]. The overall purpose of the randomized control trial is to determine the effects of a 2-year digital HC intervention on dementia risk, cognitive decline, and general health outcomes among at-risk middle-aged to older adults. General inclusion criteria include an age requirement between 45 and 75 years old, at least 2 risk factors for AD from the Australian National University-Alzheimer Disease Risk Index (ANU-ADRI), no more than 1 protective factor for AD from the ANU-ADRI, possession of a smartphone, and the ability to read and understand English. General exclusion criteria include the presence of a mental health or neurologic condition, dementia or related impairment, and current participation in a formal cognitive training or lifestyle change program.

A convenience sampling recruitment approach was used for this study with a target sample size of 200 participants to ensure adequate statistical power and up to 20% attrition. Potential participants were recruited from the northwest region of Arkansas through advertising on National Public Radio, advertising on a university daily newswire service, social media, and word of mouth. Individuals expressing interest in the study were emailed a link to an inclusion or exclusion survey instrument that was used to determine whether they were an eligible candidate for study enrollment. Each participant agreed to be randomly assigned into either the HC intervention or health education (HE) control group prior to any assessments being completed. As part of the study, participants completed a demographics survey, a series of cognitive assessments (eg, Neurotrack’s Digital Cognitive Battery), physical evaluations (eg, Short Physical Performance Battery), and biometric measures (eg, APOE status) in a laboratory setting. The HC group received asynchronous messages 1-2 times per week from their health coach with health education articles on various lifestyle modification based on the participant’s goals (eg, increase physical activity). The HC group also had unlimited access to their coach for questions and recommendations. Once a month, the participants met with their coach via videoconference or phone to discuss their goals. Coaches were required to have an initial college degree in a health- or wellness-related field (ie, nutrition, kinesiology, and psychology) as well as being a national board–certified health and wellness coach. Additionally, all coaches went through an internal Brain Coaching program developed by a team of scientists and neuropsychologists. Full criteria and study details are reported in the study’s protocol [12].

### Ethical Considerations

The study was approved by the University of Arkansas Institutional Review Board (2009280813). Informed consent was obtained from all participants in accordance with the ethical standards of Helsinki. The data were deidentified prior to analysis. Participants were compensated US $100 for this portion of the study.

### Participants and Procedures

In total, 103 participants were assigned to the HC group. Participants were 97% (n=100) White, 73% (n=75) female, with an average age of 64.2 (SD 7.6) years. All participants were assigned a personal health coach with sessions taking place remotely through monthly videoconferences and weekly asynchronous chat messages. The intervention focused on improving brain health through the following lifestyle domains and their associated risk factors: nutrition, physical activity, sleep, stress, social engagement, and cognitive activity. Recommendations were tailored to fit the needs of the participants who can reach out to their coach as often as they need. They also received supplemental resources including workout routines, healthy recipes, and health education articles.

### Data Analysis

Qualitative data were collected at the 4-month reassessment time point (time point 1) in 2021. The questions were created by the research team to explore the participants’ general perception of the HC intervention. Feedback was collected from a total of 80 of the 103 (77.7%) participants via email. The findings from the open-ended questions, “What were your top 1 or 2 takeaways from your recent Health Coaching session?” and “Is there anything you would change about our Health Coaching sessions?” were analyzed by JRM, KNB, and JM using the traditional method of thematic analysis [21]. This method was selected to ensure accurate review and interpretation of the participants’ feedback. All 3 researchers read through the data with JRM and JM creating general codes and a codebook. An inductive approach was implemented with open coding, developing codes from the raw data. Each open-ended response was coded, and through analysis and interpretation the codes were assigned to categories and larger themes using QSR NVivo software (version 9; QSR International Pty Ltd) and manual coding. The codes were reviewed and revised for clarity and removal of redundant codes. Upon triangulation and consensus, the final themes were confirmed.

## Results

### Overview

Four core themes emerged from the participants’ feedback: the importance of developing healthy lifestyle behavioral strategies, a sense of self-awareness through introspection, the value of coach support, and desired changes in program structure (Table 1). These themes encompass the spectrum of attitudes and acceptability of the intervention in our sample. Each theme is described in greater detail below with corresponding illustrative participant quotes with participants referred to as “participant” followed by a number. Names and any other identifying information were removed.

**Table 1:** Qualitative themes and definitions of rural-residing participants’ initial perceptions of the digital health coaching sessions for dementia risk at time point 1 (4 months).

Theme	Definition
The importance of developing healthy lifestyle behavioral strategies	The value of goal setting and strategies for healthy lifestyle and behavioral change
A sense of self-awareness through introspection	Self-reflection of thoughts, feelings, and motives for lifestyle choices
The value of coach support	Positive perceptions of guided support from coach
Desired changes in program structure	A desire for longer or more frequent sessions with more structured formatting

### Theme 1: The Importance of Developing Healthy Lifestyle Behavioral Strategies

As expected, most of the participants mentioned strategies for goal setting and behavior changes relative to their targeted lifestyle focus area (eg, diet, exercise, and stress) as a takeaway from their coaching sessions. These included reframing their perspective on health, understanding the value in goal setting and habit formation, as well as aligning their health goals with their values. Goal setting is a useful strategy to help individuals implement positive behavioral changes. Research has shown that goal setting can help individuals reduce risk factors associated with dementia [22]. For the participants, recognizing the importance of aligning their goals with their values and setting realistic expectations may foster greater motivation and goal achievement long term.

Importance of addressing barriers to achieving health goals. Importance of aligning values with goals.

Participant #101

That real progress can be made with little steps, the 1% idea. 1% improvement is actually a big deal, especially when it comes to health.

Participant #257

That it is time to shift away from a weight loss focus into a focus on lowering my cholesterol

Participant #265

I’ve got the tools to reach my goals. I have to resist the easy path until new habits are set.

Participant #228

### Theme 2: A Sense of Self-Awareness Through Introspection

Self-awareness through introspection was another major theme identified from the participants’ responses. Many participants indicated that their coaching session allowed them to self-reflect on their health and life in general. Through introspection—or examining one’s thoughts, feelings, and motives—individuals can become more self-aware, paying more attention to barriers that may prevent them from engaging in healthy lifestyle habits (eg, negative self-talk). As a result, an individual can gain the insight they need to develop a plan that reduces the triggers of maladaptive lifestyle choices and promotes more positive strategies. In the context of our study, participants may feel better equipped to create achievable health goals that can lead to positive behavioral changes and overall improvement in their well-being.

Probably to not judge or criticize myself but to embrace my thoughts, reactions, emotions.

Participant #163

Try to appreciate my gifts. Also try to appreciate where I am in the world, not where I wish I were.

Participant #193

I need to work on my bias for action, rather than spending so much time in my head planning what I’m going to do for my health.

Participant #231

### Theme 3: The Value in Coach Support

Health coaches are instrumental in the success of an intervention program as they are the point-person for participants and serve as a guide and advocate for their progress. This is particularly important in priority communities where individuals are disproportionately affected by health issues and are less likely to engage in effective self-management of their conditions [23]. Participants in our sample emphasized feeling supported during this process and how the coach was able to help them discover their vision of optimal health and identify specific strategies for achieving their goals.

[My coach] shares great ideas after listening to my thoughts. She never bashes or discounts what I say. She finds the good in everything. The practical suggestions are great!

Participant #329

[My coach] is always ready to support me in positive ways and strategize with any difficulties. Also really like the resources [they] find for me to use with exercise and diet.

Participant #144

[My coach] is a good listener and seems to care about my wellbeing.

Participant #255

During and after our sessions I’m thoughtful about the topics of our discussions. [My coach] is encouraging and helpful and I appreciate that. She never sounds critical or impatient. She suggested online links that might help me to sleep better. And, reinforces my goal to eat healthier by sending links to recipes.

Participant #167

### Theme 4: Desired Changes in Program Structure

It is important to note that 93% (74/80) of participants in this sample indicated that no changes were needed to the program. Individuals who indicated that changes were needed expressed the desire for a longer program, more meetings, and better program design. As feedback is early in the intervention, long-term attitudes concerning the program structure will be valuable for assessing outcomes.

Longer! I appreciate that right now we can sign up for an additional session this month.

Participant #212

From my personal experience, coaching should take place often and more consistently to be effective. Once every week or every other week is necessary for true change and accountability. I no longer work with my diet/lifestyle coach, but I met with her one-on-one weekly for a year.

Participant #305

Have a process to provide recommendations about risk factors prior to coaching sessions – provide more structured discussion rather than coach starting with ‘what are your goals’ – this project is different than routine coaching, where someone chooses to hire a coach with a specific goal in mind.

Participant #159

## Discussion

### Principal Findings

The purpose of this study was to use open-ended feedback data to explore the attitudes and acceptability of a digital cognitive HC program among rural-residing individuals at risk for AD. In doing so, we used participants’ responses to assess their initial perception of a digital HC intervention. Participants described that takeaways from the program included (1) the importance of developing healthy lifestyle behavioral strategies; (2) a sense of self-awareness and introspection; (3) the value of coach support; and (4) the desire for changes in program structure. These results illustrate the prospect for a digital HC lifestyle intervention to be well received among at-risk groups and are supported by similar findings in the literature [19-24].

As research has shown a disconnect between intervention technologies and the relationship between providers and patients [25], our results suggest the opposite—an enhanced relationship between the participants and their health coach. Additionally, in the themes that emerged from the analysis, it was evident that the initial perception of the intervention was overwhelmingly positive due to the participants’ relationship with their health coach and the personalized interactions. Even the desire for the program to be longer or more frequent by some of the participants (theme 4) highlights the perceived value of coach support (theme 3) and the potential impact on their ability to achieve and maintain their health goals. Without coach support, it is not clear how confident participants would initially feel in their ability to develop and implement healthy lifestyle behavioral strategies (theme 1) or reflect on their thoughts and emotions as it relates to their role in their health (theme 2). As a result, inadequate coaching support could affect participants’ long-term adherence and engagement overall.

### Implications

Previous research has shown that rural residents prioritize personal connections and educational opportunities for a virtual health coaching experience to have a significant impact [19]. In this study, we were able to provide further support for this notion, as many participants commented on the positive impact that their coaching experience had on their ability to live a healthier lifestyle as well as feeling a personal and positive connection with their coach. Further research is needed to determine whether these positive coaching experiences persist throughout the intervention. Several lifestyle habits have been identified as risk factors for an elevated risk of developing dementia, such as excessive smoking or drinking alcohol, lack of physical activity, and poorer diet [5]. Long-term lifestyle changes to these habits may help to reduce the risk of developing dementia, and research shows that HC can improve many lifestyle factors associated with a higher risk of dementia [9,10,26,27]. In this study, many participants commented on the change they were able to make in various different lifestyle domains; some participants’ feedback discussed the specific lifestyle changes they were able to make (eg, modifying their diet or increasing physical activity), whereas other feedback was more general and discussed successful goal setting and achievement thanks to the coaching support. Moreover, many participants commented on their newly found self-awareness because of the coaching program and reflected on their desire to continue to work on improving various lifestyle domains. As previously mentioned, this investigation was part of the ongoing DCMARVel project. Another paper out of this project demonstrated that among the same health coaching participants who adhered to the digital HC program, there was a significant improvement on their ANU-ADRI protective score from baseline to their 4-month follow-up [28]. Taken together, these results and the positive feedback in our study support the idea that a coaching program helps at-risk individuals to create positive changes in the dementia-related lifestyle risk factors. However, additional research is warranted to investigate if an HC program can reduce an individual’s risk for dementia. The use of virtual health care provides more access to these services for many individuals who may have otherwise had minimal or lack of access, such as those living in a rural community. In this study, we investigated virtually administered HC for those who are at risk of developing dementia and living in a rural midwest community. Our results showed that participants’ initial feedback was overwhelmingly positive toward the coaching program, which demonstrates the promising use of a digitally administered HC program for this population.

### Limitations

Despite best efforts to recruit a diverse sample through multiple methods of recruitment, the majority of participants were White women. As the project was limited to a localized rural community, generalizability of the results is restricted. The participants were also volunteers and were made aware of the study’s purpose prior to providing their informed consent to participate; therefore, those who chose to participate may have had a higher level of independent functioning or access to resources in the community. Taken together, a less homogeneous study in other priority communities is warranted. The digital literacy of the participants was also not assessed and may have impacted the participants’ perception of the intervention (eg, ability to use teleconferencing tools). However, it should be noted that none of the participants’ feedback referenced any concerns or issues related to digital literacy. Last, while the results are promising, a more robust collection of data via open-ended questions to gather more information about the participants’ experiences is warranted for further exploration of their perspectives and overall acceptability.

### Conclusions

The findings showed that a digital HC lifestyle intervention for cognitive health was well received by participants. Given the reception and acceptability of a digital intervention among a rural sample, the use of technology may help to address barriers of accessibility to health services in rural communities. By expanding the capacity and reach of such services, early detection and intervention can help reduce risk factors associated with AD. Furthermore, the positive feedback about their health coach offers insight into the importance of the coach-participant relationship and may serve as a significant factor in overall participant success regarding the utility of healthy lifestyle strategies and changes. Additional research is underway investigating the efficacy of the intervention program on AD risk reduction.

## Abbreviations

AD: Alzheimer disease
ANU-ADRI: Australian National University-Alzheimer Disease Risk Index
APOE: apolipoprotein E
DC-MARVel: Digital Cognitive Multidomain Alzheimer Risk Velocity
FINGER: Finnish Geriatric Intervention Study to Prevent Cognitive Impairment and Disability
HC: health coaching
HE: health education

## Data Availability

Due to the ongoing nature of the parent study, the data discussed in this study are not publicly available. However, aggregate data specific to this study are available from the corresponding author by reasonable request.
